# Occupational Stressors and Access to COVID-19 Resources among Commuting and Residential Hispanic/Latino Farmworkers in a US-Mexico Border Region

**DOI:** 10.3390/ijerph19020763

**Published:** 2022-01-11

**Authors:** Annie Jane Keeney, Amy Quandt, Mercy D. Villaseñor, Daniela Flores, Luis Flores

**Affiliations:** 1 School of Social Work, San Diego State University, San Diego, CA 92182, USA; mvillasenor9882@sdsu.edu; 2 Department of Geography, San Diego State University, San Diego, CA 92182, USA; aquandt@sdsu.edu; 3 Imperial Valley Equity & Justice Coalition, Calexico, CA 92231, USA; d309flores@gmail.com (D.F.); jr.luisf@gmail.com (L.F.J.)

**Keywords:** Hispanic/Latino farmworkers, US-Mexico border, migrant stress, mental health, COVID-19, precarious workers

## Abstract

Hispanic/Latino and migrant workers experience high degrees of occupational stress, constitute most of California’s agricultural workforce, and were among the most impacted populations by the COVID-19 pandemic. However, relatively little is known about the occupational stress experienced by farmworkers who commute daily between the US and Mexico. Occupational stress is considered an imbalance between the demands at work and the capabilities to respond in the context of the workforce. The goal of this study is to determine the type and severity of stressors in daytime and resident farmworkers and how COVID-19 vaccination status contributes to these stressors. Interviews containing the Migrant Farmworker Stress Inventory (MSWSI) were administered to a sample of 199 Hispanic/Latino farmworkers in Imperial County, a multi-billion-dollar agriculture sector in the US. Principal factor analysis differentiated latent factors in the MFSWI. Simple linear regression models and correlations identified associations between MFWSI scores and sample characteristics. The MFWSI reduced to five stressor domains: Health and Well-Being Vulnerabilities, Inadequate Standards of Living/Unknown Conditions of Living, Working Conditions, Working Environment, and Language Barriers. Approximately 40 percent of the respondents reported significant stress levels, with foreign-born (*p* = 0.014) and older respondents (*p* = 0.0415) being more likely to experience elevated stress regardless of their nighttime residence. We found that Spanish-language COVID-19 outreach might have been particularly effective for workers who reported high stress from English-language communication (*p* = 0.001). Moreover, our findings point to the importance of worker and human rights to mitigate the high-stress foreign-born workers who live in Mexico and the US experience.

Farmworkers are the foundation of the agricultural sector in the United States, providing the labor that feeds the US and beyond. However, both historically and now amid a global pandemic, these essential workers continue to have worse health and safety outcomes than other occupations in the United States. Referred to as “America’s winter salad bowl”, Imperial County produces more than 100 commodities, including two-thirds of the vegetables eaten during the winter in the United States [1]. Imperial County is geographically unique, where farm workers can reside on both sides of the US-Mexico border. In addition to various work-related stressors, farmworkers in Imperial County were hard-hit during the COVID-19 pandemic. The COVID-19 mortality rate in Imperial County is more than double that of the second-highest county in the state, and early research has found that high worker distress and large household sizes contributed to the infection and spread of COVID-19 in the region [2,3,4]. (Therefore, critical efforts are needed to improve the health and safety of farm workers in Imperial County, regardless of which side of the border they reside.

The main objective of our study is to compare daytime and resident Imperial County Hispanic/Latino farmworkers and analyze how widespread occupational stressors intersect with COVID-19-related stressors. This study is crucial in addressing the safety and health of daytime and resident farmworkers in the US-Mexico border region. The results provide insight and preliminary data to inform the development of responsive outreach strategies specific to public health efforts, including current and future vaccination efforts to ensure the health and safety of one of the region’s most vulnerable workforce.

## 1. Background

The COVID-19 pandemic exacerbated the social inequities that exist between different groups of workers. Essential (non-health) occupations, specifically workers in industries such as manufacturing, transportation, construction, food, and agriculture, were considered vital for maintaining the health and safety of the nation. However, the occupational risk of COVID-19 exposure, COVID-19 infection, and poor mental health outcomes have been found to be greater among low-wage and precarious workers in non-health essential occupations [5,6]. Recent data from the Washington State Department of Health found that agriculture, forestry, fishing, and hunting employees tested positive for COVID-19 at higher rates than any other employment category after health care and social assistance [7]. In California, Chen and colleagues [8] found that sectors with the highest relative and per capita excess mortality were among transportation/logistics workers (91 excess deaths per 100,000), facilities workers (83 excess deaths per 100,000), and food/agriculture workers (75 excess deaths per 100,000). Furthermore, across racial and ethnic groups, Hispanic/Latino working-age Californians experienced the highest relative excess mortality, the highest excess mortality among Hispanic/Latino workers being in food/agriculture (97 per 100,000) [8].

In 2018, California was responsible for producing two-thirds of the United States’ fruits and nuts and one-third of the country’s vegetables; Imperial County alone generated an estimated $2 billion of agricultural sales [9]. However, many farmworkers are undocumented, lack health insurance, and do not qualify for unemployment insurance or federal COVID-19 relief, placing much of California’s estimated agricultural workforce of 420,000 at risk [10]. In California, Hispanics/Latinos accounted for 95 percent, and immigrants accounted for 85 percent of COVID-19 deaths among farm workers in the state [11]. Imperial County has the highest proportion of non-white residents in California and ranks the highest among California’s 58 counties for social and economic factors such as injury deaths, income inequality, unemployment, and children living in poverty [12,13]. Agriculture is Imperial County’s largest industry, and the health outcomes of those working within the sector should be of particular importance, including those workers whose nighttime residence is not stateside. Commuting from Mexicali, Baja California, Mexico, the daytime farmworker population is typically in the United States daily from 2–3 a.m. to 4–6 p.m. to ensure that Imperial County’s food supply chain continues. Yet, these workers remain essentially invisible to county public health efforts and are excluded from vital safety net programs. 

Even before the COVID-19 pandemic, farmworkers were vulnerable to high stress levels, leading to substance abuse, poor mental and physical health conditions, and high injury rates [14,15,16,17,18,19]. The National Institute for Occupational Health and Safety (NIOSH) developed the National Occupational Research Agenda (NORA) for occupational health and safety research [20]. One research priority in the NORA for Agriculture, Forestry, and Fishing is “Reduce the risk of illnesses and injuries in vulnerable worker populations”, including migrant and foreign-born workers [20]. One explanation given for why foreign-born migrant workers have worse health and safety outcomes is that they experience “precarious employment”, or in other words, lack employment security [21]. Lack of security regarding employment and even housing can result in workers’ refusal to speak out about unsafe working and living conditions, take more significant risks, and perform tasks without the necessary safety equipment [21]. Psychological distress correlates with precarious employment [22].

Fielding-Miller and colleagues [23] found that as of 12 July 2020, a higher percentage of farmworkers, residents living in poverty, higher density, higher population, and a higher percentage of residents over the age of 65 were all independently and significantly associated with a higher number of deaths in a county. The COVID-19 crisis has only exacerbated the burdens of precarious work among Imperial County farmworkers. Between April 2019 and April 2020, Imperial County saw an 81% increase in agricultural-related job loss [3]. Imperial County ranks third among California counties in the incidence of “high worker distress”, a measurement of households living below a living wage with larger than average household sizes [24]. (). Moreover, Kerwin and Warren [25] report that migrant farmworkers are disproportionately represented among COVID-19 victims due to most agricultural workers’ inability to socially distance or participate in any telecommuting capacity. The risk of psychological harm related to increased COVID-19 morbidity and mortality to a community already more likely to have a history of trauma and distress than the general US population can be devastating [26].

## 2. Materials and Methods

For this comparative assessment study, we sought a purposeful sample of Hispanic/Latino farmworkers working in Imperial County, California, who were at least 18 years old. The participants were informed of the study through a trusted community gatekeeper, the Imperial Valley Equity and Justice Coalition (IVEJC). The IVEJC developed a farmworker database between February and April 2021 in partnership with Imperial County healthcare providers to secure vaccine appointments for farmworkers. The database includes contact information for approximately 3600 farmworkers residing in either the US or Mexico, a random sample of 260 farmworkers were contacted by telephone, and 53 agreed to complete the survey over the phone—many did not answer. After participating in a short training about the research purpose and methods, IVEJC coalition members invited farmworkers to participate in a survey via phone call or in-person efforts. The remaining surveys were conducted through in-person efforts consisting of four site visits by IVEJC members to popular transit points for farmworkers along the US-Mexico border. Two site visits occurred at 3:00 a.m., while workers waited for transit to harvest sites, and two visits occurred at 4:00 p.m. as workers returned to Mexico at the end of their workday. Phone calls were administered to farmworkers registered in the IVEJC database. Because surveys were conducted anonymously, outside the workplace, and by a trusted community organization with an ongoing presence at farmworker transit points, we do not believe that fear of workplace retaliation influenced selection in our sample.

We collected data over three months (June 2021–August 2021) from 199 Hispanic/Latino farmworkers working in Imperial County. Most surveys were administered in Spanish by bicultural and bilingual researchers who recorded responses in real time through Qualtrics, a web-based platform. We offered a $10 gift card for the participant’s time spent answering the survey questions. Participants who completed the survey were mailed or handed the $10 gift card immediately after. The San Diego State University institutional review board approved the study procedures and instrument (Protocol Number: HS-2021-0093). 

### 2.1. Instrumentation

The Migrant Farmworker Stress Inventory (MFWSI) [27] was translated from English into Spanish and uploaded into Qualtrics. The research assistants used a non-expiring link generated by the survey platform to record responses in real time. The MFWSI is a 39-item instrument used to measure stress in migrant farmworkers [27].The respondents rate each item on a 5-point scale (“Have Not Experienced” to “Extremely Stressful”). The total MFWSI score is obtained by summing the scores for all the items; possible MFWSI scores range from 0 to 156. Higher scores mean a greater degree of stress related to the migrant farmworker lifestyle. Individual scores of 80 or more indicate that the individual may have stress levels that pose significant mental health risks. Cronbach’s analysis for the MFWSI found excellent reliability (α = 0.94), consistent with previous studies [28,29]. Using the same 5-point scale, we also sought responses to two additional questions: “I worry about the air I breathe at work is not clean” and “Sometimes I feel like I don’t get enough sleep”. Additionally, we sought demographics including age, gender, nighttime residence, birth country, commute time from home to work, number of household members, common health issues (asthma, high blood pressure, diabetes), and COVID-19-related questions such as positive test results for COVID-19 and COVID-19 vaccination status. 

### 2.2. Data Analysis 

Data collected from Qualtrics were uploaded into SPSS (v. 27) (IBM SPSS Statistics, Chicago, IL, USA) and STATA (MP 16) (StrataCorp LLC, College Station, TX, USA) for analysis. Because one of the study’s goals was to describe similarities and differences between the daytime and resident farmworker population, we used univariate analyses to explore the overall characteristics of the sample. Bivariate analyses were used to examine correlations between MFWSI scores and demographic variables. A principal axis factor analysis with varimax rotation was conducted to assess the underlying structure of the 41 items on the survey (39 items from the MFWSI plus the two added items). Based on the previous literature [29]) we believed that different stressors would “hang together” and could be reduced to five latent scores to find factors that best fit the data. Assumptions were tested and met using the Kaiser-Meyer-Olkin (KMO) and Bartlett tests, indicating that the items could be reasonability reduced to discrete factors. Factor loadings of less than 0.30 were suppressed. Loadings of 0.40 and above were examined in the context of the grouped items from each factor and were assessed to see if they fit together conceptually and could be named. Simple linear regression was conducted to see how well categorical, demographic variables predicted MFWSI scores. We did not include the two additional questions (sleep and air quality) in the MFWSI score analysis.

## 3. Results

### 3.1. Characteristics of the Study Participants

A total of 199 Hispanic/Latino farmworkers working in Imperial County participated in the study. Most respondents indicated that their nighttime residence on workdays is in the United States (62.2%), yet most respondents were foreign-born (76%—of whom 98% were born in Mexico). The overall average age of respondents was 46 years. Most respondents identified as male (*n* = 117, 59%); 77 respondents (39%) identified as female, and four respondents preferred not to say (2%). Women farmworkers were more likely to be US nighttime residents (78% of women vs. 59% of men). The average commute time for respondents (time from leaving their residence to work site) for the overall sample was just under 2 h (116.7 min). The participants whose nighttime residence is Mexico had an average commute time of 194 min, compared to 72 min for US nighttime residents. Slightly more than a quarter of the respondents shared that they had tested positive for COVID-19 (27%) within the last year, and 75% of the respondents reported receiving the COVID-19 vaccine. A higher percentage of respondents who reported having health issues (e.g., diabetes, high blood pressure, asthma) were US residents. See Table 1. 

### 3.2. Principal Axis Factor Analysis 

Principal axis factor analysis with varimax rotation was conducted and produced a 5-factor solution. After rotation, the first factor accounted for 14.1% of the variance, the second factor accounted for 12% of the variance, the third factor accounted for 9.4% of the variance, the fourth factor for 6.3%, and the fifth factor accounted for 3.3%. Table 2 displays the items and factor loadings for the rotated factors, with loadings of less than 0.4 omitted to improve clarity. The first factor, “Health and Well-Being Vulnerabilities”, contained six items reflecting interpersonal or physical conditions that US-Mexico border farmworkers experience (Cronbach’s alpha (α) = 0.84). The second factor, “Inadequate Standards of Living/Unknown conditions of living”, included nine items reflecting feelings of social vulnerability and how unknown conditions of living resulting from farm work can impede obtaining an adequate standard of living (α = 0.84). “Working Conditions” consisted of five items about the lack of social, emotional, and physical protection related to the occupation of farm labor (α = 0.80). The “Working Environment” factor included four items reflecting concerns the farmworkers have while working on the field site, particularly among other workers (α = 0.78). Finally, the fifth factor, “Language Barriers”, consisted of two items assessing how communicating in English (e.g., not native language) can affect an individual’s perceived stressors associated with working (α = 0.70). The amount of variance accounted for by these factors ranged from 3.3% to 14.1% and combined accounted for 45% of the variance in the survey.

### 3.3. Migrant Farmworker Stress Inventory (MFWSI) Scores

**Figure 1:**
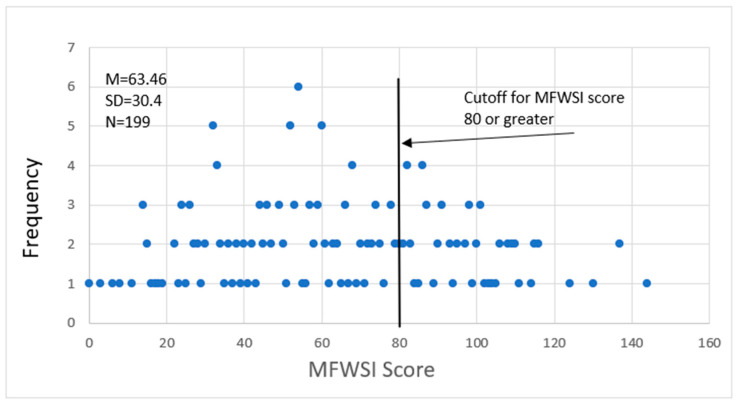
Relative frequencies of respondents’ MFWSI scores.

Participant scores on the MFWSI ranged from 0 (no stressors present) to 144 (significant stressors experienced) as shown in Figure 1. Note that the maximum MFWSI stress score is 156. The MFWSI scores showed that 75 participants (38.5%) reported substantial stress levels, with a score of 80 or higher. Farmworkers in this category were more likely to be born in Mexico (42% vs. 22%, χ^2^ = 6.0019, *p* = 0.014) and older (49 years vs. 45 years on average, *p* = 0.0415). The average stress score among residents and commuting farmworkers was 63.5, with an average score of 65 for participants who indicated their nighttime residence stateside and an average score of 60 for those who indicated their nighttime residence as Mexico (not statistically significant). The average stress score was statistically significantly different between respondents born in Mexico (67 on average) and those born in the United States (52 on average) (*p* = 0.0224). Non-significant demographic variables included gender, nighttime residence, education, received COVID-19 vaccine, and commute time. 

Logistic regression was conducted to assess whether age and country of birth significantly predicted whether a participant had a stress score of 80 or more. The assumptions of observations being independent and independent variables being linearly related to the log were checked and met. When both variables are considered together, they significantly predict whether a participant will have a stress score of 80 or more, x^2^ = 5.81, df = 2, N = 193, *p* < 0.05. 

### 3.4. Stressors for Daytime and Residential Farmworkers

The top five stressors for the overall sample were: (1) “Sometimes I don’t get enough sleep”, (2) “It is difficult to be away from family members”, (3) “I have to work in bad weather”, (4) “I worry about not having medical care” and (5) “I have to work long hours”. Two of the top five stressors varied by nighttime residence. Mexican nighttime residents were more likely to experience stress from “Sometimes I feel like I don’t get enough sleep” (χ^2^ = 11.2095, *p* = 0.024), while US nighttime residents were more likely to experience stress from “I have to work in bad weather”, and “I worry about the air I breathe at work is not clean” (χ^2^ = 9.8893, *p* = 0.042). See Table 3 for stressors by nighttime residence. 

### 3.5. Nighttime Residence, MFWSI Stressors, and COVID-19 Variables

COVID-19 positive test result and vaccination status were not significantly correlated with nighttime residence. For example, 69% of US and 67% of Mexican nighttime residents reported not testing positive for COVID-19. Vaccination rates were also similar, with only 21% of US and 30% of Mexican nighttime residents reporting having not received any doses of a COVID-19 vaccine. 

To investigate if there was a statistically significant association between stressors inherent in farm work and COVID-19 vaccination status, Pearson’s correlations were computed. Receiving the COVID-19 vaccine and difficulty in communicating in English were significantly correlated. The direction of the correlation was positive, meaning that participants who had received the COVID-19 vaccine tended to report having more stress related to communicating in the English language, r(195) = 0.25, *p* = 0.001. Using Cohen’s [30] guidelines, the effect size is small to medium for studies in this area. Receiving the COVID-19 vaccine and not having medical care was approaching statistical significance, r(197) = 0.13, *p* = 0.068. 

To investigate whether there was a statistically significant association between stressors inherent in farm work and COVID-19-positive results, Pearson’s correlations were computed. Stressors associated with “I have to work long hours” and “It bothers me people use drugs” were significantly correlated with testing positive for COVID-19. The direction of the correlations was positive, which means that the participants who reported testing positive for COVID-19 tended to have higher experiences of stress associated with long work hours, r(180) = 0.16, *p* = 0.03, and others’ drug use, r(180) = 0.16, *p* = 0.032. Using Cohen’s [30] guidelines, the effect size is small for studies in this area. 

## 4. Discussion

Our research examines the implications of COVID-19 infections and vaccine access on known occupational stressors of farmworkers in Imperial County, comparing farmworkers who reside on both sides of the US-Mexico border. Our analysis produced three main findings:

1. Despite similar social and cultural characteristics, the results provide novel insights into how daytime and resident farmworkers experience stressors. We found no differences in COVID infections and vaccine access between the US and Mexico nighttime residents.
2. The results show that age and country of birth (i.e., foreign-born) variables can predict greater stress levels associated with the farmworker lifestyle than nighttime residence. This finding supports research on the enduring impact of experiences of citizenship, language, and cultural barriers, as foreign-born workers residing in Mexico and the US were both more likely to report elevated levels of stress.
3. We found that the MFWSI (including the two additional items we added) reduced to five latent factors of stressors confronting US-Mexico border region farmworkers: health and well-being vulnerabilities, inadequate standards of living/unknown conditions of living, unfavorable working conditions, working environment, and language barriers. Moreover, the top five stressors experienced by respondents were related to sleep, family, working conditions, and working environment.

More importantly, this study increases our understanding of how fundamental worker and human rights are needed to support precarious workers’ overall health and safety in the agricultural sector. Prior to the COVID-19, in 2019, the International Labour Organization Centenary Declaration asserts “*safe and healthy working conditions are essential for decent work*” [31] (p. 60). Occupational justice is particularly significant today. Ensuring the health and safety at work, specifically among low-wage and non-health-essential occupations, is imperative to effective pandemic management, public health prevention efforts, and protecting a highly vulnerable workforce [5,6].

It has been established that foreign-born farmworkers often experience poor mental health and, most recently, are disproportionately affected by COVID-19, including increased mental health challenges and stress associated with the loss of family members and reduced income and work, and lack of personal protective equipment [32].. The results of this study complement and extend the research on Hispanic/Latino farmworker health and mental health. Among Hispanic/Latino farmworkers working on the US-Mexico border, there are several differences or lack thereof between commuting and residential workers that are particularly noteworthy:

- Mexican nighttime residents were more likely to experience stress from “Sometimes I feel like I don’t get enough sleep”. By contrast, US nighttime residents were more likely to experience stress from “I have to work in bad weather” and “I worry about the air I breathe at work is not clean”. Given that participants whose nighttime residence is Mexico had a commute of 194 min, compared to 72 min for US nighttime residents, a lack of sufficient sleep for Mexico nighttime residents may be related to having much longer commute times.
- Women were more likely to be US resident workers, indicating that family composition and wage earner responsibilities could influence employment opportunities and residential choice.
- The higher reported chronic health conditions among US-based workers might reflect a difference in medical access (compared to Mexico-based workers) and gender in health-seeking behavior.

Research has demonstrated that women are more likely to seek care and visit their primary care physician to a greater extent than men for physical and mental health concerns [33]. Given that most US-based workers in the study were women, access to medical knowledge (of one’s chronic conditions) may raise awareness of exposure to unhealthy environments. In contrast, those with less understanding of their chronic conditions are more concerned about familial and immediate physical (lack of sleep) sources of stress. Moreover, there are cultural beliefs among Hispanic/Latino individuals that going to the doctor is only necessary when one is sick, impacting preventive education and intervention [33]. Lastly, the lack of a statistical difference in COVID infections is particularly noteworthy since there was much concern in the region that daily migrants would be more exposed to COVID-19 due to clustering at the international crossing, waiting for buses together, and then busing together to work sites. There was an assumption in the region that US resident workers would have a lower risk of infection from safer commutes. These findings suggest that COVID-19 infections may be more likely to happen at the worksite, perhaps due to prolonged close contact and lack of personal protective equipment, both of which have been found in previous studies [25,32].

On the MFWSI scale, an individual score of 80 or more indicates stress levels that pose significant mental health risks. Our study found that approximately 40% of the respondents reported significant levels of stress. Farmworkers in this category were more likely to be foreign-born, regardless of nighttime residence and, on average, older. We found that nighttime residence was not a significant predictor of stress levels among respondents. Given the fluidity of the US-Mexico border in this region, this is particularly interesting. Unpredictable border closures and cross times in response to the COVID-19 pandemic force workers to make decisions about work (e.g., access to wages) and activities of daily living (e.g., where to sleep, transportation, access to food) if their nighttime residence was inaccessible. Yet, these circumstances did not influence the levels of stress among participants. However, farmworkers born in Mexico reported significantly higher stress levels. These findings correspond with studies that indicate foreign-born, Hispanic/Latino farmworkers experience high levels of trauma, distress, anxiety, and depression [15,34,35,36]. Furthermore, death by suicide is the second leading cause of intentional injury incurred by foreign-born workers in the United States [37]. Given the overall poor health outcomes and healthcare (including mental health) professional shortages in Imperial County, our findings suggest a concern for suicide risk among Imperial County Hispanic/Latino farmworkers.

Studies have indicated that most people who die by suicide have a mental or emotional disorder, with an estimated 30% to 70% experiencing depression or bipolar disorder [38]. For male migrant farmworkers in North Carolina, Hiott and colleagues [29] found that working conditions were strongly linked to depression symptomology. Our research extends this concern further. We found that the MFWSI (including sleep and air quality items) reduced to five latent domains of stressors confronted by US-Mexico farmworkers: health and well-being vulnerabilities, inadequate standards of living/unknown conditions of living, working conditions, working environment, and language barriers. Our findings suggest that US-Mexico farmworkers, perhaps given the unique geographical location, experience precarious occupational stressors differently than migrant farmworkers working in other areas of the US. For example, Hiott et al. [29] found work permits and deportation as stressors associated with a legality and logistics factor. However, these items did not “hang” with any of the reduced factors in our analysis, suggesting perhaps that the fluid nature of the US-Mexico border region is more conditioned to accept/ignore documentation status. Although documentation status is presumably stressful for migrant workers, our findings suggest that this was more illustrated in the factor “inadequate standards of living/unknown conditions of living”. Inadequate wages, difficulties associated with finding a place to live, reliable transportation, consistent employment, and access to social services were the stressors confronted by our respondents. Lack of worker rights and adequate social supports are often associated with undocumented precarious workers [21,22,34,39,40,41] Furthermore, stressors associated with the border (e.g., closures, delays) and increased national attention on immigration policy may suggest that US-Mexico border farmworkers experience stressors associated with lack of job security at a higher rate than those that cannot easily cross the border. 

Conditions are typically associated with how a person physically or mentally feels, whereas environment can mean the immediate physical surroundings of that person, including nature, people, and climate. These nuances were most pronounced in the “unfavorable working conditions” and “working environment” factors that were derived from the data. Of interest was that our findings indicated that others’ substance use (alcohol and drug use) “hung” together with respondents’ concerns of air quality and bathroom conditions, suggesting that substance abuse was a visible part of the physical work environment. Studies have demonstrated that substance rates are lower in Mexico than in the US [42,43]; however, migration can lead to increased substance abuse [43,44]. In a study conducted 10 years before the legalization of recreational marijuana, Sanchez-Huesca and colleagues [43] found that the most consumed drugs among migrant workers are cocaine and crack (56.5%) because of the energy provided, methamphetamines (26.1%) as they allow users to stay awake and work longer, and marijuana (26.1%) because of its relaxing effect. Given our study’s context of daily migration and legalized recreational marijuana, there is an increased concern not only for US -Mexico border farmworkers’ substance use, but also for the secondhand effects of substance abuse that can contribute to overall unsafe and unhealthy work environments at agricultural field sites. Additional research on the secondhand effect of substance abuse within farming populations is warranted. 

While our results complement other studies that highlight language barriers as an occupational stressor [15,29] and most recently are demonstrated to be barriers to accessing COVID-19 resources, vaccination appointments, and testing among Hispanic/Latino farmworkers [45], our findings also suggest the effectiveness of community-based partnerships for supporting and disseminating vital public health information to a population often excluded and underserved. Difficulty communicating and understanding others in English were identified stressors for respondents. Of interest is that we found a statistically significant positive correlation between difficulty communicating in the English language and confirmed COVID-19 vaccination status. Many survey respondents in our sample were previously contacted by IVEJC through outreach efforts, including a Spanish-language hotline, aimed at mitigating the linguistic, technological, and informational barriers that prevented farmworkers from registering through the standard vaccine registration portals. It seems likely that callers to the Spanish-language IVEJC vaccination hotline experienced high stress from communicating in English. This might explain our finding that as the respondents’ stress levels increase due to difficulties communicating in English, the likelihood of reporting COVID-19 vaccination also increases. 

Our findings have several practice and policy implications. Some stressors could be easily improved upon, and it is critical that local farming associations advocate for efforts to reduce stressors within their control. For example, advocating for improving or increasing bathroom conditions and capacities in the field could improve the health and safety of farmworkers. Bathroom condition stress was in the top third of stressors for all respondents and is a realistic stressor employers could address that could have immediate positive implications. Furthermore, it may behoove employers to consider how to support drug and alcohol use among hired farmworkers, not only from a health and safety standpoint but also from a sustainable business model. Substance abuse can impact employers’ bottom line when lost productivity, absenteeism, turnover, healthcare, disability, and workers’ compensation are accounted for in cost expenditures. Within the agriculture industry, it is estimated that the per capita costs to employers for each untreated worker with a substance use disorder is $2,689 [46]. Given the scale of the regions’ agriculture operations and the required workforce needed to maintain operations, cost savings could be in the billions. For example, in 2000, the estimated cost of lost productivity for Texas farmworkers due to alcohol and drug use was $11.2 billion [47]. The reluctance to health-seeking and the exclusion from vital safety net resources among farmworkers put agriculture employers in a critical position to make meaningful improvements in the lives of their workforce. We encourage employers to take steps to support substance abuse resources and treatment proactively. By doing so, employers in the US-Mexico border region may see a reduction of the secondhand effects of substance abuse, thus further creating a safer, healthier, and more productive work environment that has the potential to increase profits significantly. 

There are clinical implications as well. When community-based organizations can build trust and connect with US-Mexico border farmworkers, for instance, ensuring access to COVID-19 vaccinations, these can be excellent times to screen for depression, anxiety, or substance abuse. These opportunities are critical in disseminating safety and health information and connecting farmworkers to necessary behavioral health resources. 

### 4.1. Future Research 

The novel findings from this study inform additional areas to explore and understand the intersection of occupational stressors and COVID-19 related stressors on mental health outcomes. We suggest conducting studies that aim to assess the levels of stress that can come from COVID-19 and working conditions. For example, stress related to accessing personal protective equipment, colleagues’ behavior towards mask-wearing and social distancing guidelines, and concerns about contracting COVID-19 from the workplace and bringing COVID-19 home to loved ones would be essential to explore among Hispanic/Latino farmworkers. Understanding the degree of these stressors will further help scholars understand how stressful job conditions and individual and situational factors influence the risk of injury and illness in a COVID-19 era. Moreover, extending these inquiries to a larger sample of rural Hispanic/Latino workers could help inform the development of culturally responsive strategies to better support occupational justice, health, and safety efforts.

Additionally, it appears that mental health remains a low priority in the region, as indicated by one mental health provider per 660 residents, which is 2.4 times higher than the state average ratio [12].In 2018, the average number of mentally unhealthy days reported in the past 30 days for Imperial County was 4.7, higher than the state average of 3.7 [12]. Given that increased mental health concerns, suicidal ideation, and substance abuse have been reported in response to the COVID-19 pandemic [48], we can anticipate that the pandemic has only aggravated the behavioral mental health concerns in Imperial County. The World Health Organization asserts, “to deal with the burden of mental [illness] and reduce the psychosocial vulnerabilities of individuals, attention needs urgently to be paid to the determinants that can be modified of the development, onset, progression, and outcome of mental problems” [49] (p.17). Occupational justice and work-life well-being must be considered as one of these determinants. We must recognize how cultural differences, subjective judgments, and theories related to mental health are defined among Hispanic/Latino farmworkers. Future studies should aim to explore these definitions. 

### 4.2. Limitations

Despite the potential implications of our findings, it is important to acknowledge three significant limitations of the methodology used in this study. First, the survey was conducted between June and August, while due to the extremely hot temperatures in Imperial County, most vegetables are harvested between January and June. This means that there may be segments of the farmworker population who were not working during this time and thus not reached, particularly during our in-person survey efforts. However, drawing from the vaccine registration database constructed between February and April mitigates this limitation to a degree. Second, the survey included only quantitative questions about predetermined stressors from the MFWSI. This means that there may be other vital stressors faced by farmworkers in Imperial County that are not included in the MFWSI. We attempted to mitigate this limitation by adding air quality and sleep items to the survey instrument that we know are pertinent to farmworkers in the US-Mexico border region. Third, because IVEJC’s farmworker database is primarily composed of individuals who were assisted in registering for COVID-19 vaccines, there may be a bias toward vaccination status in our sample. This makes it difficult to estimate vaccination rates among the broader farmworker population from our study. We mitigated this limitation in our data by conducting some in-person surveys at farmworker transit points.

## 5. Conclusions

This study compares occupational stressors and COVID-19 infections/vaccine access between daytime and resident farmworkers in a US-Mexico border region. Importantly, these two farmworker populations had similar rates of positivity for COVID-19, as well as vaccination rates. Significant stressors were associated with the working environment and inadequate standards of living/unknown conditions of living. Instead of nighttime residence, country of birth (i.e., foreign-born) and age were predictors of greater MFWSI scores, indicating that the individual may have stress levels that pose significant mental health risks. Results complement and extend the research on Hispanic/Latino farmworker behavioral and mental health and provide novel insights into the occupational stressors that farmworkers experience in a unique geographical region. The fluidity between the Mexico and US border afforded to farmworkers in the area may suggest an increased risk for severe behavioral and mental health concerns, particularly around substance abuse and the secondhand effects of substance use.

Understanding how to reduce stressors in the immediate working environment is a pertinent issue for the agricultural safety and health of the region.

We specifically hope to see policy changes that improve workers’ rights regarding bathroom conditions and address the secondhand effects of substance abuse in the field because of this study. A significant positive correlation between difficulty communicating in English and being vaccinated for COVID-19 illustrates the impact of community-based partnerships in mitigating language barriers and improving trust in public health efforts. We feel that agricultural safety and health stakeholders that partner with community-based organizations could have a meaningful impact on increasing access, awareness, and availability of behavioral health services for the US-Mexico border farmworker population.

## Figures and Tables

**Table 1:** Personal characteristics of Hispanic/Latino farmworker study participants (*n* = 199).

Personal Characteristic	N	%
Age Mean (SD)	46.38 (15.0)	- -
No. of persons living with you Mean (SD)	3.7 (1.8)	- -
No. of persons living with you who are also farmworkers Mean (SD)	1.68 (1.6)	- -
Commute Time (in minutes) Mean (SD)	116.7 (116.1)	- -
Country of Birth (*n* = 198)		
US	48	24.2
Mexico	147	74.2
Peru	1	0.51
El Salvador	2	1.01
Gender Identity (*n* = 198)		
Male	117	59.0
Female	77	38.9
Prefer not to say	4	2.02
Nighttime Residence (*n* = 193)		
US	120	62.2
Mexico	65	33.7
US/Mexico	8	4.2
Highest Level of Education (*n* = 199)		
No Formal Schooling	5	2.51
Elementary	28	14.07
Middle	71	35.68
High School	69	34.67
University/College	16	8.04
Technical School	2	1.01
Some College	8	4.02
Health Conditions		
Diabetes	22	11.1
Prediabetes	18	9.0
High Blood Pressure	46	23.1
Asthma	11	5.5
Tested Positive for COVID-19 (*n* = 193)		
Yes	53	26.6
No	131	65.8
I don’t know	9	4.5
Did you receive COVID-19 vaccine (*n* = 197)		
Yes	148	74.4
No	49	24.6

**Table 2:** Factor loading for the rotated factors.

Item	Factor Loadings				
	1	2	3	4	5
**Factor 1: Health and well-being vulnerabilities**					
My life has become more difficult because my partner is no longer with me (because he or she has moved or has died	0.792				
I worry about who my children are spending time with	0.640				
I have health problems because of physical work	0.601				
There are no stores nearby	0.560				
I do not get enough credit from other family members for the work I do	0.521				
I worry about my relationship with my partner	0.509				
**Factor 2: Inadequate Standards of Living/Unknown conditions of living**					
Sometimes I have difficulty finding a job		0.606			
I find it difficult to talk about my feelings to other people		0.551			
It is difficult to complete the paperwork necessary to receive social services		0.549			
Sometimes I have difficulty finding a place to live		0.514			
Sometimes I don’t feel at home.		0.511			
At times I have not been able to buy things that I want because I make little money		0.489			
Migrating to the United States was difficult		0.482			
I sometimes worry because I do not have reliable transportation		0.479			
Sometimes I feel my housing in inadequate		0.452			
**Factor 3: Working Conditions**					
I have to work long hours			0.631		
It is difficult to be away from family members			0.628		
Sometimes I don’t feel settled			0.581		
I work in bad weather			0.547		
I worry about not having medical care			0.486		
**Factor 4: Working Environment**					
It bothers me that other people drink too much alcohol				0.660	
It bothers me that other people use drugs				0.508	
I worry about the air I breathe				0.484	
Sometimes I feel that the conditions of the bathrooms are bad				0.434	
**Factor 5: Language Barriers**					
Difficulty communicating in English					0.692
I have difficulty understanding other people when they speak English					0.477
% of Variance	14.1%	12%	9.4%	6.3%	3.3%

**Table 3:** Top five stressors by nighttime residence.

Stressor	United States M (SD)	Mexico M (SD)	US/Mexico M(SD)
Sometimes I feel like I don’t get enough sleep	3.06 (1.2)	3.22 (1.2)	3.71 (0.76)
It is difficult to be away from family members	3.14 (1.9)	2.77 (1.2)	3.38 (1.2)
I have to work long hours	2.98 (1.2)	2.62 (1.2)	3.5 (0.92)
I work in bad weather	3.06 (1.2)	2.92 (1.2)	3.25 (1.2)
I worry about not having medical care	2.72 (1.3)	2.60 (1.3)	3.13 (1.4)

## Data Availability

Restrictions apply to the availability of these data. The data are not publicly available due to IRB protocol and procedures.
